# Liquid and Pressure-Sensitive Adhesives Based on Cassava Starch and Gelatin Capsule Residue: Green Alternatives for the Packaging Industry

**DOI:** 10.3390/foods12213982

**Published:** 2023-10-31

**Authors:** Yuliana Monroy, Sandra Rivero, María Alejandra García

**Affiliations:** 1 Centro de Investigación y Desarrollo en Criotecnología de Alimentos (CIDCA-CONICET), 47 y 116 S/N, La Plata B1900AJJ, Buenos Aires, Argentina; monroyuliana92@gmail.com (Y.M.); sandragmrivero@gmail.com (S.R.); 2 Facultad de Ciencias Exactas, Universidad Nacional de La Plata (UNLP), 47 y 115 S/N, La Plata B1900AJJ, Buenos Aires, Argentina

**Keywords:** biobased adhesives, rheological behavior, citric acid, adhesive capacity

## Abstract

Natural polymer-based adhesives are *green* alternatives, necessary to reduce the problems impacted by synthetic adhesives. Starch and gelatin have extraordinary potential for the synthesis of biobased adhesives. Citric acid (CA), a natural acid, induces the crosslinking and hydrolyzing of both gelatin and starch. In this sense, this work deals with the use of gelatin capsule residues as a promising material to produce biobased adhesives in combination with cassava starch in the presence of different CA concentrations characterizing their mechanical, physicochemical and microstructural properties. Depending on CA concentration, formulations adjusted to different applications can be obtained such as liquid and pressure-sensitive adhesive films. The inclusion of CA allows us not only to improve the applicability of the system since it modifies the flowability of the adhesives as evidenced by the observed changes in the viscosity (from 158.3 to 90.3 for formulations with 20 and 80% CA, respectively). In addition, mechanical profiles showed that the inclusion of CA increased the adhesive bond strength (from 2230.7 to 2638.7 for formulations with 20 and 80% CA, respectively). Structural modifications induced by CA in adhesive formulations were highlighted by ATR-FTIR analysis.

## 1. Introduction

Greater awareness of the need to preserve non-renewable natural resources has led to the development of more ecological high-performance polymers with new functionalities [1]. In recent years, there have been increasing efforts in the adhesive industry to improve the sustainability of processes and products. This is due, on the one hand, to the interest in developing eco-compatible formulations and, on the other, the impending shortage of petroleum, from which many chemical products are derived. Natural polymer-based adhesives are “green” alternatives, necessary to reduce the problems caused by synthetic adhesives [2,3]. In the adhesive industry, this has manifested itself through the gradual shift from solvent adhesives to water-based or high-solid ones and renewed interest in adhesives based on natural polymers. Therefore, the development of eco-compatible adhesives that allow easy and quick washing is seen as an alternative with potential functionality to minimize environmental impacts, also contributing to the sustainability of the processes.

One of the alternatives for the production of biodegradable systems is the use of polymers obtained from renewable resources or waste and industrial by-products. Uranga et al. [4] reported that the recovery of biomass waste and its subsequent introduction into different production chains allow for the more efficient use of resources and contribute to the sustainability of the processes, in line with the premises of the circular economy. In this sense, biobased adhesives can be obtained from natural resources like vegetable or animal products such as starch and their derivatives, cellulose, proteins, casein, collagen and gelatin among others [5,6,7]. 

In this way, starch and gelatin have extraordinary potential for the synthesis of various formulations due to the versatility of functional groups which in turn offer unlimited opportunities to introduce modifications in their structure [6,8]. 

Currently, one interesting source of protein to develop biodegradable films is the residue generated by the industrialization of gelatin capsules, which are used to deliver bio-active compounds in the nutraceutical sector [5,9,10]. Therefore, the valorization of industrial by-products to produce bioadhesive materials may contribute to scaling down the amount of residue disposal reducing the use of non-biodegradable synthetic polymers [11].

On the other hand, starch has advantages such as being biodegradable, low cost and renewable, and it contains hydroxyl groups with strong intra- and intermolecular hydrogen bonding [7]. Citric acid is a natural acid, and it has been already demonstrated to be suitable for the crosslinking of gelatin and starch [12,13].

Adhesive formulations in the packaging industries are essential to give structure to most paper, board or glass packaging, and therefore, the design of pressure-sensitive adhesives (PSA) represents an innovative development. A pressure-sensitive adhesive is an adhesive system that is permanently tacky and adheres to a variety of surfaces [14]. To the best of our knowledge, studies related to the design of biodegradable pressure-sensitive adhesives produced from biopolymers are scarce [15]. In this sense, Sartori et al. [15] developed biodegradable pressure-sensitive adhesives produced from wheat gluten.

In addition, the incorporation of an economical and non-toxic polycarboxylic acid such as CA is proposed as an overcoming strategy, which allows for improving the system and introducing distinctive adhesive characteristics, compared to commercially available products. In line with the current needs and requirements, the novelty of the present work is focused on the design of versatile liquid and/or pressure-sensitive adhesive formulations for bonding different substrates such as paper and glass, among others, with potential applications in food packaging and/or in the labeling processes.

The aims of this research were to develop biobased adhesives (liquid or solid) from the use of gelatin capsule residues as a promising biopolymer in combination with cassava starch in the presence of different CA concentrations characterizing their mechanical, physicochemical and microstructural properties. An in-depth study of the structural modifications induced by the addition of citric acid and their correlation with techno-functional properties was carried out.

## 2. Materials and Methods

### 2.1. Formulation of Biobased Adhesives

Water-based composite adhesives were developed from mixtures of cassava starch (*Manihot esculenta*, Montecarlo, Misiones) and gelatin from discarded drug capsules (Bagó, Argentina), containing 2.91% *w*/*w* of TiO_2_. The starch–gelatin dispersions were hydrated and treated in a thermostatic bath in the presence of citric acid (CA, purity degree 99,5%) (Biopack, Argentina), for 20 min at 90 °C. The composite adhesives were formulated with 5% *w*/*w* starch and 15% *w*/*w* gelatin, to obtain a final solids concentration of 20% *w*/*w*. Citric acid was incorporated into the formulations at 20, 40 or 80% *w*/*w* based on the concentration of the polymer blend [16]. Nomenclature used for each system was CA-20, CA-40 and CA-80, respectively.

#### 2.1.1. Rheological Characterization

Rheological analyses were performed in a G2 TA Instrument (New Castle, DE, USA) rheometer using a plate–plate sensor of parallel standard steel plates of 40 mm at controlled temperature (20 °C) with a gap of 1 mm. Tests of the viscoelastic behavior of the different adhesives were carried out in dynamic mode. First, stress sweeps (0–100 Pa) were performed at constant frequency (1 Hz) to determine the range of linear viscoelasticity. Frequency sweeps (0.01 to 100 Hz) were then performed at a constant stress value (1 Pa). The dynamic rheological parameters recorded were the storage modulus (G′), the loss modulus (G″) and the tangent of the offset angle (tan δ = G″/G′). Mechanical spectra were obtained by plotting G′ and G″ as a function of frequency (ɷ). Rheological tests were performed at least in duplicate.

#### 2.1.2. Back Extrusion

Formulation consistency, as well as their changes during storage time, was studied by the back extrusion technique in a TA-XT2i texturometer (Stable Micro Systems, Surrey, UK) at 25 °C [17]. The back extrusion rig P/75 probe was used.

### 2.2. Adhesive Capacity of Liquid Formulations

The ASTM D6195–03 (2019) standard procedure was adapted to evaluate the tacking capacities of formulated bioadhesives. A loop of Kraft paper 25 mm wide with a minimum length of 175 mm was formed and held with the A/TG tension clamp probe in a TA-XT2i texturometer. The paper loop was placed in contact (25 × 25 mm contact area) with the other substrate (paper or glass) containing the adhesive layer to be evaluated which was kept fixed on the base of the equipment and peeled off at a speed of 5 mm min^−1^. Tests were carried out on 8 replicates at least.

Likewise, formulations were applied forming a thin and uniform adhesive layer on the paper strips, as described in a previous work [18]. The roughness of the samples (Kraft paper or glass) was determined by using a PCE-RT 1200 roughness tester (Schwyz, Switzerland). The parameters of roughness were expressed as the average roughness (Ra) and the mean peak-to-valley height (Rz), [19].

Peel strength tensile tests (*peel T* assays) were performed in a TA.XT2i—Stable Micro Systems (Surrey, UK) texturometer, using A/TG uniaxial tensile grips at a test speed of 10 mm s^−1^ [18]. Kraft paper specimens (at least 10 per sample) were prepared according to ASTM F904 with a bond area of 50 mm × 25 mm (paper or glass). 

### 2.3. Analysis of the Microstructure by ATR-FTIR and SEM

The effect of CA concentration on the adhesive formulation was studied by ATR-FTIR and SEM. Fourier transform infrared spectroscopy (FTIR) spectra were recorded in Nicolet iS10 Thermo Scientific equipment (Madison, WI, USA). Films of each formulation, obtained by drying in an oven at 37 °C, were placed on the diamond crystal accessory to obtain FTIR spectra combined with attenuated total reflectance (ATR). Likewise, the native cassava starch suspension, gelatin capsules and CA were also analyzed. Spectra were obtained (as the average of at least 32 scans) between 4000 and 400 cm^−1^, with a resolution of 4 cm^−1^, which were analyzed with baseline correction using OMNIC™ software (version 8.3, ThermoFisher Scientific, Waltham, MA, USA).

The morphology of the bioadhesive films was studied by SEM using a Quanta200 FEI model electron microscope (Eindhoven, The Netherlands). The accelerating voltage used was 20 kV, under low-vacuum conditions. In order to study the microstructure of adhesive films, the samples were bonded to bronze blocks with double-sided carbon tape, which allowed visualization of the surface and its cross-section.

### 2.4. Contact Angle

Contact angle measurements were made in a model 190 Goniometer (Ramé-Hart, Instrument Co., New Jersey, USA) on substrates with bioadhesives after drying. For this, a Kraft paper specimen was placed on a slide, which was fixed to the support with double-sided tape, to avoid any waviness in the paper. A uniform layer of adhesive was applied to the cellulosic substrate and then dried at room temperature. For the determination, milliQ water was used in the syringe of the goniometer, and at least 10 measurements were performed on each sample [18]. 

### 2.5. Evaluation of the Adhesive Capacity of Pressure-Sensitive Formulation

The combination starch: gelatin: citric acid allowed us to obtain a film with self-adhesive properties whose nomenclature is CA-80. The adhesive (100 g) film was performed using 24 cm diameter silicone molds and dried at 37 °C for 24 h. The films were unmolded, and 2 × 4 cm specimens were cut; then, the adhesion was tested on different surfaces: ceramic, metal, glass, plastic, paper, cardboard and Durlock^TM^. Adhesion was studied using the cross-cut test following the ASTM D3359 [20] standard, with slight modifications. This is a standardized test that allows qualitative estimation of the adhesion of a coating on a substrate using a test tape. It is a widely used, fast and inexpensive visual comparison method for evaluating the adhesion of film coatings to metal substrates by applying and removing a pressure-sensitive tape over grid cuts in the film.

In accordance with the standard, the adhesive film was cut into small squares (2 × 2 mm), and to evaluate the adhesion according to ISO 2409 and ASTM D 3559-B [20], a standard test method for measuring adhesion by tape test was used. To make the cut, a sharp tool was used to obtain a cross-cut of 36 squares. A commercial adhesive (3M^TM^ 3903 and Double A^TM^, recognized for their adhesive properties on a wide variety of substrates) tape was placed over the traced grid which was then quickly pressed and removed. The adhesion of the adhesive film was determined according to the classification criteria of images and descriptions based on the aforementioned standard.

### 2.6. Statistical Analysis

All experiments were performed at least in duplicates. Analysis of variance (ANOVA) and comparison of means with Fisher’s least significant difference (LSD) test were conducted, at a significance level of α = 0.05. The InfoStat Software, version 2020 (InfoStat Group, Agricultural Sciences College, National University of Cordoba, Cordoba, Argentina), was used. 

## 3. Results and Discussion

### 3.1. Rheological Behavior of Composite Adhesive Formulations

Composite bioadhesives formulated with cassava starch and gelatin chemically modified in the presence of different concentrations of citric acid were obtained, taking advantage of the functionality and capacity of citric acid to modify these polymers. 

The gelatinization of cassava starch in the presence of underutilized gelatin and citric acid allowed obtaining polymeric blends with pseudoplastic behavior. All the adhesive formulations had a white appearance due to the TiO_2_ coloration contained in the underutilized capsules of the pharmaceutical industry which presented acidic characteristics with pH values below 4. The pH decreased with the CA content, with values close to 4 for samples formulated with 20% *w*/*w* CA, in the range of 2–3 for bioadhesives prepared with 40% *w*/*w* CA and 1–2 for the maximum concentration of the CA tested (80% *w*/*w*). Regarding the stability of the adhesives over time, no syneresis (obvious sign of starch retrogradation) was observed even after one week of storage at room temperature, which demonstrates the degree of stability of the developed adhesives. In this sense, it is important to stress that adhesives formulated without CA solidify and are unmanageable, and, in addition, they do not have the ability to stick.

**Figure 1:**
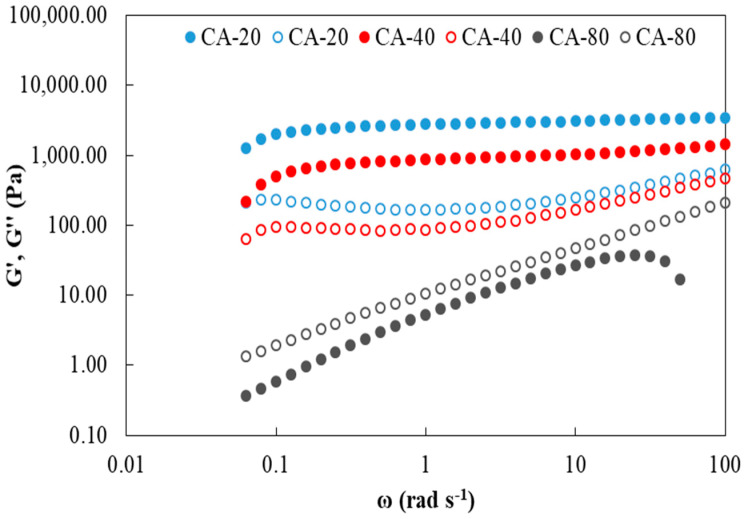
Effect of the citric acid concentration on the evolution of the elastic modulus (G′, filled circles) and viscous (G″, empty circles) of bioadhesives based on cassava starch and gelatin. Nomenclature used: CA = citric acid, the following number (20, 40, 80) indicates the concentration of CA incorporated into the adhesive % (g acid/100 g of polymer mixture).

Dynamic-mechanical spectra of the composite adhesive formulations are shown in Figure 1. The bioadhesives exhibited a behavior dependent on the citric acid content. Figure 1 shows a typical spectrum of a gel for the samples containing 20 and 40% *w*/*w* CA, where the elastic modulus (G′) was higher than the viscous one (G″) throughout the frequency range analyzed.

Firoozmand et al. [21] reported that gelatin achieves maximum elasticity by lowering the pH below its isoelectric point (pI = 4–4.5). Likewise, the authors pointed out that in a composite matrix, the pH value influences the degree of compatibility and the nature of the interactions established between the biopolymers, which affects the microstructure and viscoelastic properties of the resulting gel. In accordance with Goudie et al. [22], a dense and compact structure was promoted at a pH close to the isoelectric point, where the interactions within the gelatin network are equilibrated. 

On the other hand, with the addition of 80% CA, the rheological behavior corresponds to that of a concentrated solution (Figure 1). This could be explained by the hydrolysis that gelatin undergoes at the working pH (1–2). According to Wang et al. [23], citric acid hydrolyzes gelatin, and its effect increased with the CA concentration leading to a formulation that weakened its gelling capacity.

In addition, consistency studies were conducted to complement the rheological analysis. Table 1 shows the values of the parameters obtained for the different adhesive formulations developed. From the analysis of the back extrusion test results obtained for the biobased formulations, the effect of citric acid is evident (Table 1). The firmness and consistency of the adhesive decreased significantly (*p* < 0.05) at high concentrations of CA, indicating its predominantly hydrolyzing action on the polymer mixture. A similar trend was observed for the cohesivity and viscosity index.

### 3.2. Adhesive Capacity of Composite Formulations

The development of adhesive formulations was proposed for their potential use in gluing cellulosic and glass substrates, thinking about their subsequent application in the bottle packaging and/or labeling area. 

**Figure 2:**
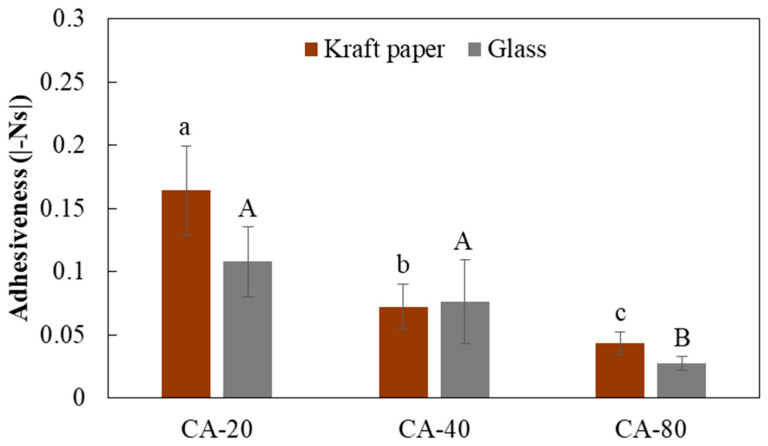
Adhesiveness determined from loop adhesion tests of the developed formulations as a function of the CA concentration. Different letters (lowercase for Kraft and uppercase for glass) indicate significant differences (*p* < 0.05) for each substrate studied.

Tack is the ability of an adhesive to wet the surface and adhere to the substrate and depends on the mobility of the adhesive’s constituents and their ability to wet the substrate. When evaluating peel strength, external pressure is applied to allow complete wetting of the adhesive on the substrate [24]. Loop adhesion testing is routinely performed on commercial adhesives and is used for the quality control of adhesive tapes and pressure-sensitive adhesives to evaluate the tacking. The adhesive formulations registered a decrease in adhesiveness and so in stickiness when the acid concentration increased (Figure 2), in close relationship with the evolution of the rheological characteristics evaluated through the back extrusion test (Table 1). Kumar et al. [25] reported that CA is an excellent crosslinking agent that favors the interaction between citric acid carboxyl groups and starch hydroxyl groups.

There was evidence of a significant decrease (*p* < 0.05) in adhesiveness with the concentration of CA (Figure 2). The difference in values between the substrates was mainly due to the roughness of each one of them. Dohr and Hirn [26] pointed out that the roughness of the adherent surface strengthens the bond with the adhesive by increasing the bond area and promoting mechanical interlocking. The glass presented a value of Ra = 0.04 ± 0.01 μm and Rz = 0.3 ± 0.04 μm while the Kraft paper registered Ra and Rz values of 3.1 ± 0.4 μm and 30.1 ± 0.8 μm, respectively. At higher roughness, the adhesive can penetrate the structure of the substrate and improve the adhesiveness and characteristics of the adhesive–substrate bond. Therefore, characteristics of the substrate such as its greater roughness and porosity are factors that promote greater adhesion. It is necessary to ensure a good wettability of the substrate by the adhesive, since the cavities not reached by it constitute potential points of initiation of rupture of the adhesive bond.

When an adhesive is formulated, it is intended to provide an adhesive bond of adequate strength. This means that the failure mode is not intended to occur at the substrate–adhesive interface but rather that this interface is as strong as the substrate [27]. In this sense, while adhesive and cohesive failure is undesirable, since it indicates a weak adhesive bond, substrate failure indicates that the adhesive bond is strong, reaching the maximum strength of the material [26]. 

**Figure 3:**
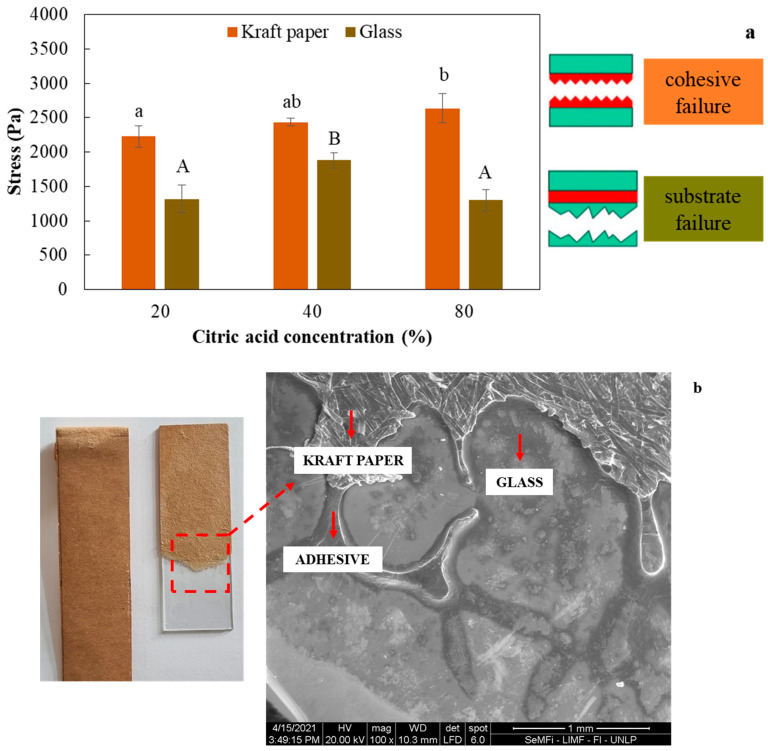
(**a**) Effect of citric acid concentration (CA, % *w*/*w*) on the bonding strength of different substrates (Kraft paper–Kraft paper or Kraft paper–glass). Different letters (lowercase for Kraft and uppercase for glass) indicate significant differences (*p* < 0.05) for each substrate studied; (**b**) SEM micrographs of the adhesive–substrate interface after *T-peel* test.

The mechanical adhesive behavior of the different formulations developed for bonding cellulosic Kraft paper substrates is shown in Figure 3. With CA concentration, stress values of Kraft paper samples bonded with the bioadhesive did not change significantly (*p* > 0.05). Meanwhile, tensile *peel* tests on Kraft paper samples bonded to glass showed a variation with the CA concentration, with the CA-40 formulation being the one that showed a significant increase (*p* < 0.05) in the stress values (Figure 3).

Regardless of the bioadhesive formulation, compatibility was observed between the system under study: glass–adhesive–Kraft paper, which could benefit from the interactions with the silanol groups that make up the glass and the carboxylic groups of the bioadhesives, in addition to possible interactions by hydrogen bonding that occurs between the cellulosic material and the adhesive (Figure 3b).

All the formulations exhibited filmogenic capacity (the ability to form a film by drying), and the film obtained only with the formulation with 5% starch-15% gelatin and 80% citric acid/100 g of polymer mixture (P1-80) proved to have self-adhesive capacity.

As is known, both the surface tension of the adhesive and the surface energy of the substrate affect the contact angle. The adhesive’s hydrophobicity showed a significant increase (*p* < 0.05) with CA concentration in the formulation (Table 2). These results could be attributed to the crosslinking effect of CA (at 40 and 80%) that favors the crosslinking of the polymeric mixture during heat treatment, which leads to obtaining matrices with hydrophobic characteristics.

### 3.3. Structural Modifications Induced in Adhesive Formulations: ATR-FTIR Studies

The structural modification of the starch–gelatin system in the presence of CA was studied using the ATR-FTIR technique.

From the gelatin capsule (G) spectra, the characteristic absorption peaks of the amide band I (corresponding to the stretching vibration of the carbonyl C=O) and the amide II band (corresponding to the bending vibration of the C-N-H bond) were detected at 1627 and 1530 cm^−1^, respectively. Similar results were reported by He et al. [28]. Furthermore, the absorption peak at 1240 cm^−1^ was assigned to the amide III band, corresponding to the C-N stretching vibration. All the bioadhesives exhibited a broad band located at 3500–3000 cm^−1^ ascribed to the free and bound -NH and -OH absorption groups capable of forming hydrogen bonds with both gelatin and starch [4]. 

**Figure 4:**
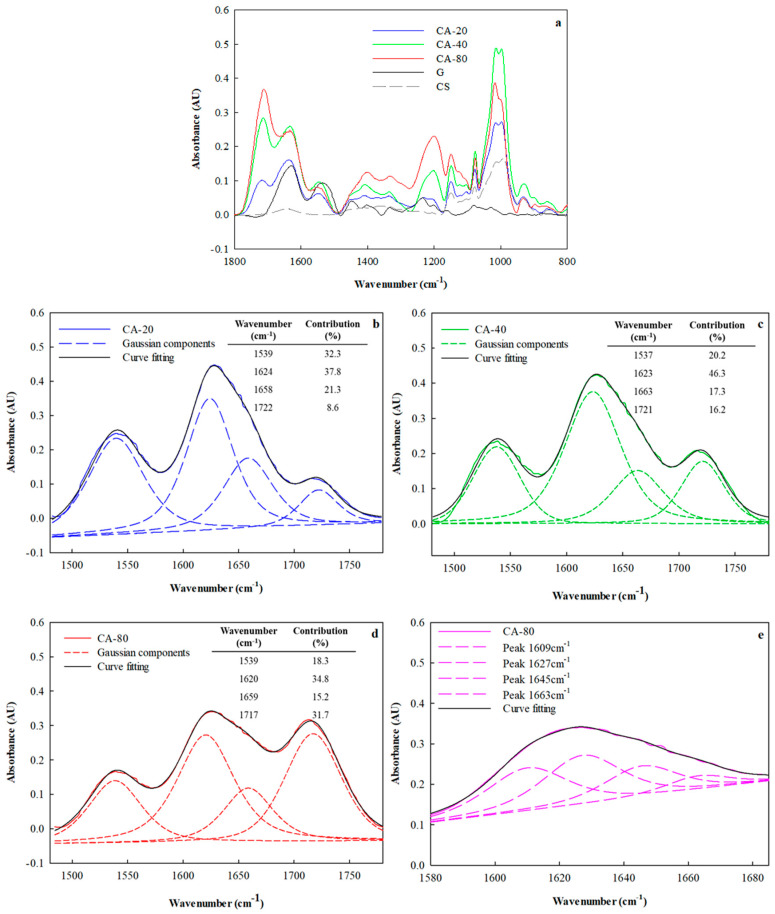
(**a**) ATR-FTIR spectra of adhesive films formulated in the presence of CA at 20%, 40% and 80% *w*/*w* (g acid/100 g polymer mixture). Second derivative curves in the spectral region from 1450 to 1850 cm^−1^ for adhesive containing different CA concentrations: (**b**) CA-20, (**c**) CA-40 and (**d**) CA-80; and (**e**) second derivative curves in the spectral region from amide I for the sample CA-80. Experimental data (solid colored lines), individual components of the Gaussian (dotted lines) and curve fitting (solid black lines).

All bioadhesives exhibited the broad band at 3500–3000 cm^−1^ adsorbed by free and bound -NH and -OH groups capable of forming hydrogen bonds with both gelatin and starch. On the other hand, the band ascribed to the symmetric and asymmetric stretching vibration of methylene (-CH_2_) was identified at 2850 and 2919 cm^−1^, respectively. The addition of CA was correlated with the appearance of a peak at 1710 cm^−1^, corresponding to the C=O stretching vibration as a result of the interactions that it establishes with the components of the adhesive formulation. Likewise, this peak was magnified with the increase in CA concentration (Figure 4a). Similar results were reported by Kumar et al. [29] who studied biodegradable films based on modified corn starch and gelatin. In addition, Reddy and Yang [30] and Brandelero et al. [31] reported that with the inclusion of citric acid in starch-based matrices, the appearance of a band at 1724 cm^−1^ could be elucidated, corresponding to the association between the carboxyl and hydroxyl groups. In addition, Kumar et al. [29] demonstrated that citric acid esterified the hydroxyl groups of starch, inducing strong interactions between the polymer chains. In addition, this interaction could also occur between citric acid and hydroxyl groups present in gelatin. Similar assignments were made by He et al. [28] and Kumar et al. [29] for composite systems based on gelatin and starch.

Due to its functional groups (-NH_2_, -OH, -COOH/-COO−), gelatin is able to interact through hydrogen bonding and electrostatic interactions, further improving the compatibility and chemistry of the resulting systems [32]. Bands at 1646 cm^−1^ and 1363 cm^−1^ corresponded to the γ-dicarbonyl and C-N stretching of the amide groups, respectively [33].

Another difference detected from the spectra against the addition of CA is related to the changes observed in the band at 1638 cm^−1^, which would indicate that a chemical association between the system components and citric acid was established. According to Singh and Maitra [34], these changes are correlated with modifications in the configuration of the glycosidic ring as a consequence of the association of citric acid groups with starch.

Spectral deconvolution was performed to improve the resolution of the spectra, and in this way, it was possible to identify the exact position of the peaks in the ATR-FTIR spectra (Figure 4). In the adhesive formulations, a more marked change in the relative contribution of the peak located at 1620 cm^−1^ was observed with increasing citric acid concentration, confirming modifications in the secondary structure, in coincidence with that reported by Liguori et al. [12]. In contrast, a higher contribution of the peak at 1720 cm^−1^ was observed for systems with higher CA content (CA-80 vs. CA-40), which would indicate that crosslinking promoted by CA would occur in these formulations (Table inserted in Figure 4a–c).

According to Stanca et al. [35], the secondary structure components of a protein were found at different positions: β-sheet (1610–1635 cm^−1^), random coil (1635–1645 cm^−1^), α-helix (1645–1665 cm^−1^), β-turn (1662–1682 cm^−1^) and β-sheet antiparallel (1682–1689 cm^−1^). As is well known, the second derivative of the ATR-FTIR spectra is a tool that allows magnifying some spectral features that appear as weak. Spectral deconvolution was performed to ensure a more accurate estimation of the amide I component bands. From curve fitting, four Gaussian components of the amide I band in the ATR-FTIR spectrum were detected (Figure 4e). The peak assignment and the percentage contributions are shown in Table 3. CA concentrations changed their relative contributions substantially. 

According to Koochakzaei [36], after exposure to acid addition, the triple helix structure of gelatin loses its conformation. Therefore, the conversion of α-helix to β-sheet due to stability is another exchange due to changes in the secondary structure of the gelatin.

These results revealed that the CA-80 film exhibited self-adhesive properties not only to the touch but also to the aforementioned substrates. A possible explanation for these effects would be related to the conformational changes that are revealed in the gelatin in the presence of CA, evidenced by ATR-FTIR, as a consequence of which functional groups are exposed that would be responsible for the adhesive characteristics to the touch of the gelatin. A similar hypothesis was proposed by Sartori et al. [15] to explain the adhesive capacity of gluten-protein-based adhesives.

### 3.4. Pressure-Sensitive Adhesive: Characteristics and Potential Applications

**Figure 5:**
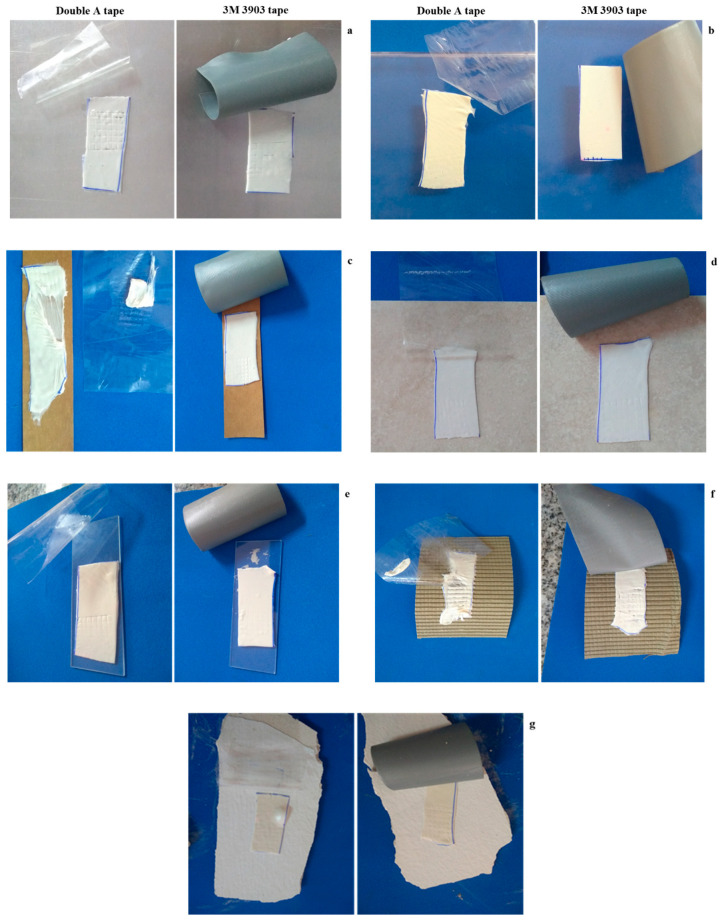
Photographs taken during the adhesion test on different substrates: (**a**) metal, (**b**) plastic, (**c**) Kraft paper, (**d**) ceramic, (**e**) glass, (**f**) corrugated cardboard and (**g**) Durlock^TM^.

In order to investigate the possibility of using the adhesive film on different substrates, a test tape adhesion assay was carried out. The CA-80 adhesive film showed a greater adhesion on the metal (Figure 5a) and plastic (Figure 5b) sheets. Both Double A^TM^ and 3M^TM^ test tapes showed no peeling of any film grid, indicating their high adhesion to these substrates. According to the ASTM D3359-02 [20] standard, the adhesion of the CA-80 film is classified as 5B, since the corners of the cuts are intact, that is, no square of the film has come off.

When evaluating the Kraft paper, it was observed that part of the bioadhesive film was dragged by the Double A^TM^ tape (Figure 5c). According to the specifications of the standard, the adhesion to this substrate is classified as 1B; the adhesive film was folded and peeled at all intersections and most of the squares, revealing an affected area of 35 to 65%.

In the ceramic (Figure 5d) and in the glass (Figure 5e), the bioadhesive film was adhered to both substrates, and the tapes could not lift the adhesive. In corrugated cardboard, the Double A^TM^ tape was again able to lift part of the CA-80 film (Figure 5f), in which the adhesive–cellulose-fiber interaction is less than that of the adhesive–test tape. This could be due to the low compatibility with the substrate and the poor penetration of the adhesive into materials. Finally, the Durlock^TM^ test was performed (Figure 5g) to evaluate its potential use as a self-adhesive film, considering possible applications as a wall adhesive, and again, the strong adhesive capacity of the studied bioadhesive film to adhere to this substrate was observed, with different characteristics from those mentioned above.

Thus, the results obtained through the standardized test, although qualitative, indicate the potential and versatility of the bioadhesive film for bonding different types of substrates.

**Figure 6:**
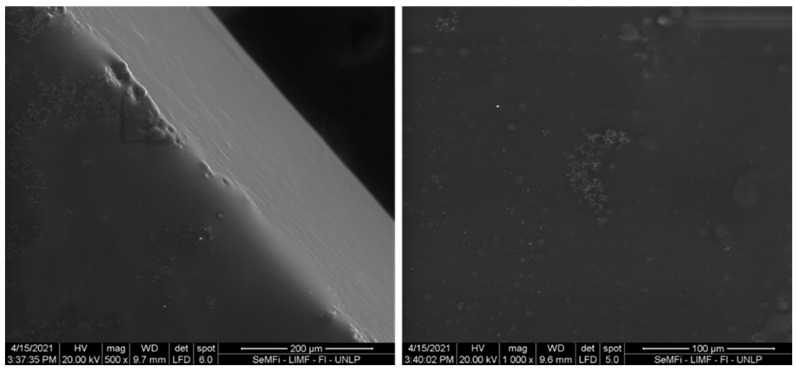
SEM micrograph of the film obtained from the formulation based on 5% *w*/*w* starch-15% *w*/*w* gelatin and 80% *w*/*w* CA (CA-80). On the left is the cross-section, and on the right is the surface. The magnification is indicated in the micrographs.

Complementary analysis by SEM could allow us to study the morphology of the pressure-sensitive adhesive. Figure 6 shows the SEM micrograph of the film obtained with the CA-80 formulation. With a compact, dense and smooth structure, the filmogenic capacity developed by the formulation can be observed, which is expected to have a positive impact on the adhesive characteristics and on the resistance to sticking of different substrates.

## 4. Conclusions

Composite adhesive formulations were developed using starch, gelatin and citric acid by heat treatment under controlled conditions. Depending on the concentration of citric acid, formulations adapted to different applications can be obtained. The inclusion of citric acid not only improves the applicability of the system by modifying the flowability of the adhesives, as evidenced by the observed changes in consistency/viscosity, but also provides increased adhesive bond strength.

In the case of liquid adhesive formulations, the results revealed that the functional properties of the starch–gelatin blends were significantly improved by the addition of citric acid up to a level of 40%. The structural changes induced by the addition of CA were revealed by ATR-FTIR analysis and allowed explaining the adhesive behavior of the different formulations. 

On the other hand, the highly adhesive properties of the material obtained by drying the CA-80 formulation revolutionize the studies and invite us to further deepen the analysis of pressure-sensitive adhesives, opening up possible industrial applications. In this sense, it is important to highlight that although liquid adhesive formulations containing 80%CA have low adhesion and tack, once dried, they allow a self-adhesive tape to be obtained. This bioadhesive film, based on chemically modified natural polymers (cassava starch and gelatin), is able to bond substrates of different nature (cardboard, paper, glass, Durlock™, ceramic, plastic).

Therefore, by tailoring the formulation of the adhesive based on cassava starch and gelatin, it was possible to obtain different types of adhesives, from liquid adhesives for Kraft paper and glass widespread use in the packaging industry to a self-adhesive tape, demonstrating the versatility of the formulations.

## 5. Patent

Monroy Y., Rivero S.M.G, García M.A. (2022). Composiciones adhesivas ecocompatibles y película adhesiva bifaz, y procedimientos de elaboración. Acta Nº 20220102305.

## Figures and Tables

**Table 1:** Back extrusion profile parameters of biobased starch–gelatin composite adhesives.

Samples	Firmness (g)	Consistency (g s^−1^)	Cohesiveness (|g|)	Viscosity Index (|g s^−1^|)
CA-20	32.1 ± 0.6 ^b^	279.4 ± 2.5 ^b^	17.2 ± 0.5 ^b^	158.3 ± 2.3 ^c^
CA-40	30.5 ± 1.3 ^b^	262.3 ± 10.9 ^b^	16.4 ± 0.3 ^b^	137.8 ± 5.1 ^b^
CA-80	22.9 ± 1.0 ^a^	215.4 ± 4.7 ^a^	13.2 ± 0.4 ^a^	90.3 ± 3.6 ^a^

Mean values ± standard deviation are presented. Different letters within the same column indicate significant differences (*p* < 0.05).

**Table 2:** Contact angle of adhesive formulations with 5% *w*/*w* starch-15% *w*/*w* gelatin and different CA concentrations on Kraft paper.

CA-20	CA-40	CA-80
76.7 ± 2.6 ^a^	81.3 ± 3.3 ^b^	85.9 ± 3.7 ^c^
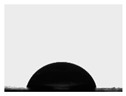	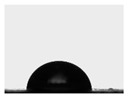	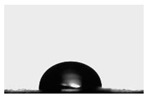

The reported results correspond to the mean ± standard deviation. Different letters indicate significant differences (*p* < 0.05). CA: 20, 40 and 80 correspond to % CA.

**Table 3:** Secondary structure contribution (%) obtained by deconvolution of the amide I region taking into account the Gaussian components of the ATR-FTIR spectra.

Wavenumber (cm^−1^)	Amide I—Contribution (%)		
	CA-20	CA-40	CA-80
1609	4.7	17.7	32.9
1626	46.5	45.9	36.5
1646	43.3	21.4	21.7
1664	5.5	14.9	8.8

## Data Availability

The data presented in this study are available on request from the corresponding author.
